# The Salivary Microbiome Is Altered in Children With Eosinophilic Esophagitis and Correlates With Disease Activity

**DOI:** 10.14309/ctg.0000000000000039

**Published:** 2019-05-20

**Authors:** Girish Hiremath, Meghan H. Shilts, Helen H. Boone, Hernan Correa, Sari Acra, Andrey Tovchigrechko, Seesandra V. Rajagopala, Suman R. Das

**Affiliations:** 1 Division of Pediatric Gastroenterology, Hepatology and Nutrition, Vanderbilt University Medical Center, Nashville, Tennessee, USA;; 2 Division of Infectious Diseases, Vanderbilt University Medical Center, Nashville, Tennessee, USA;; 3 Division of Pediatric Pathology, Vanderbilt University Medical Center, Nashville, Tennessee, USA;; 4 Research Bioinformatics, MedImmune, Gaithersburg, Maryland, USA.

## Abstract

Eosinophilic esophagitis (EoE) is an allergen-mediated inflammatory disease affecting the esophagus. Although microbial communities may affect the host immune responses, little is known about the role of the microbiome in EoE. We compared the composition of the salivary microbiome in children with EoE with that of non-EoE controls to test the hypotheses that the salivary microbiome is altered in children with EoE and is associated with disease activity.

**METHODS:**

Saliva samples were collected from 26 children with EoE and 19 non-EoE controls comparable for age and ethnicity. The salivary microbiome was profiled using 16S rRNA gene sequencing. Disease activity was assessed using the Eosinophilic Esophagitis Endoscopic Reference Score and the Eosinophilic Esophagitis Histologic Scoring System (EoEHSS).

**RESULTS:**

A trend toward lower microbial richness and alpha diversity was noted in children with EoE. Although the overall salivary microbiome composition was similar between children with and without EoE, specific taxa such as *Streptococcus* (q value = 0.06) tended to be abundant in children with active EoE compared with non-EoE controls. *Haemophilus* was significantly abundant in children with active EoE compared with inactive EoE (q value = 0.0008) and increased with the increasing EoEHSS and Eosinophilic Esophagitis Histology Scoring System (q value = 5e-10). In addition, 4 broad salivary microbial communities correlated with the EoEHSS.

**DISCUSSION:**

The composition of the salivary microbiome community structure can be altered in children with EoE. A relative abundance of *Haemophilus* positively correlates with the disease activity. These findings indicate that perturbations in the salivary microbiome may have a role in EoE pathobiology and could serve as a noninvasive marker of disease activity.

## INTRODUCTION

Eosinophilic esophagitis (EoE) is a chronic, food and/or aeroallergen-mediated inflammatory disease that affects the esophagus (1,2). It is characterized by symptoms of esophageal dysfunction (e.g., vomiting, abdominal pain, and dysphagia) and is confirmed by the presence of an intense eosinophilic inflammation (≥15 eosinophils per high-power field [eos/hpf]) in at least one of the multiple esophageal mucosal biopsies after excluding other causes of esophageal eosinophilia (3). The burden of EoE has dramatically increased since it was first described as a rare disease over 2 decades ago and is currently estimated to affect up to 57 per 100,000 individuals in the Western population (4,5).

Our understanding of the pathogenesis of EoE is incomplete. The current disease paradigm implicates both genetic and environmental factors, with environmental factors appearing to have a larger role in disease development (6–8). Among the environmental factors, the role of the commensal microbiome is of increasing interest because alterations in microbial communities can disrupt host metabolism and immune response and potentially lead to disease development (9). Furthermore, identification of microbial communities relevant to a particular disease may provide important clues regarding novel pathogenetic mechanisms and improve clinical care. At present, little is known about the role of the microbiome in EoE. To date, studies have been limited with a focus on characterizing the esophageal microbiome (10,11). Although this seems appropriate because EoE is localized to this organ, it is unclear whether the composition of the microbiota in other areas of the gastrointestinal tract such as the oral cavity, saliva, stomach, or colon is altered in this condition.

In EoE, the oral cavity is significant as a major entry point for the inciting food and aeroallergens that then interact and mix with the salivary constituents (including the salivary microbiome) and trigger an immune response that results in eosinophil-predominant inflammation in the esophagus. Therefore, from a symbiology point of view, studying shifts in the composition of the salivary microbiome may offer unique insights into pathobiology of EoE. In addition, because saliva can be collected in a noninvasive manner, identifying distinct alterations in the salivary microbiome could potentially serve as a practical and convenient approach to identify and monitor EoE.

In this study, we conducted a comparative analysis of the salivary microbiome in children with EoE vs non-EoE controls and elucidated the association between the salivary microbiota in children with EOE with EoE activity indexes.

## MATERIALS AND METHODS

### Study design and case definitions

Children aged 6–18 years either diagnosed with EoE or with symptoms of esophageal dysfunction and undergoing esophagogastroduodenoscopy (EGD) with biopsy at our center between January 2016 and November 2017 were consecutively enrolled. The exclusion criteria were subjects diagnosed with inflammatory bowel disease, celiac disease, and connective tissue disorder; a history of esophageal surgery or varices; neurodevelopmental disorders or behavioral disorders; use of systemic corticosteroids or exposure to antibiotics within the previous 30 days; or the presence of any visible oral ulcer or gingival disease observed during the bedside pre-EGD examination.

Cases consisted of children with active and inactive EoE or one or more symptoms of esophageal dysfunction and demonstration of ≥15 eos/hpf in one of the multiple esophageal biopsies after adequate proton pump inhibitor (PPI) therapy and after excluding other causes of esophageal eosinophilia. Active EoE was defined per the 2011 consensus guidelines (3,12), and inactive disease was defined as a peak eosinophil count of <15 eos/hpf. The non-EoE control group consisted of children with symptoms suggestive of esophageal dysfunction and normal esophageal histology.

### Saliva sample collection and preprocessing

Saliva samples were collected before the EGD and between 7:30 am and 11:30 am Per protocol, participants were nil per os for at least 6 hours before their EGD. Upon providing informed consent and assent, participants rinsed their mouth with 5 mL of water. After a 10-minute wait period, between 4 and 7 mL of saliva (unstimulated, whole mouth fluid) was collected as spit in a sterile tube (BD Biosciences, San Jose, CA). The samples were maintained at 4 °C and transported to the laboratory within 2 hours of collection. In the laboratory, the saliva samples were centrifuged at 3,000 rpm for 15 minutes (1,419*g*) at room temperature to remove particulate debris, and the supernatant was stored in aliquots at −80 °C until further analysis.

### Clinical, endoscopic, and histologic data

Clinical data including demographics, coexisting allergic conditions (e.g., allergic rhinitis, eczema, and asthma), recent exposure to medications (e.g., oral antihistaminics, PPIs, and nasal, inhaled, and swallowed topical corticosteroids), and dietary patterns were gathered. All EGDs were performed by a single investigator (G.H.), and any esophageal mucosal changes such as edema (0–2), rings (0–3), exudates (0–2), furrows (0–2), and strictures (0–1) were recorded per the validated Eosinophilic Esophagitis Endoscopic Reference Score (EREFS) (13). We planned to obtain 6 esophageal biopsies (3 each from the distal and proximal esophagus) to maximize EoE diagnostic sensitivity and submitted these biopsies for hematoxylin and eosin staining per standard protocol. The microscopic changes in esophageal biopsies (gold standard) were objectively assessed and graded for the intensity of eosinophilic inflammation, basal zone hyperplasia, dilated intercellular spaces, eosinophilic microabscess, eosinophil surface layering, surface epithelial alterations, dyskeratotic epithelial cells, and lamina propria thickness when present per the Eosinophilic Esophagitis Histology Scoring System (EoEHSS) by a single pathologist (H.C.) blinded to the microbiome or endoscopic data. Gastric and duodenal biopsies were evaluated for eosinophilia to exclude cases of concomitant eosinophilic gastritis or eosinophilic gastroenteritis (14).

### Sample processing, sequencing salivary microbiome, and sequence data analysis

Microbial DNA was extracted with the PowerSoil Kit (Qiagen, Germantown, MD). Microbial DNA was extracted after mechanically lysing using TissueLyser II (Qiagen) for 20 minutes. Dual-indexed universal primers appended with Illumina-compatible adapters were used to amplify the hypervariable V4 region of the bacterial 16S ribosomal ribonucleic acid gene, using polymerase chain reaction parameters as previously described (15). Each sample was run on a 1% agarose gel to verify reaction success. Libraries were cleaned and normalized with the Invitrogen SequalPrep Kit. After normalization to 1–2 ng/μL, 10 μL of each sample was combined to create the sequencing pool. The pool was cleaned with 1X AMPure XP beads (Beckman Coulter, Brea, CA). Libraries were sequenced on an Illumina MiSeq with 2 × 250 bp reads. A negative and a mock community control (ZymoBIOMICS) were run concurrently along with the samples to assess data quality and levels of background contamination. Reads were processed by following the mothur MiSeq SOP (www.mothur.org/wiki/MiSeq_SOP) as of August 4, 2017 (15). Additional details are described in the supplementary material (see Supplementary Digital Content 1, http://links.lww.com/CTG/A37).

### Statistical analysis

Descriptive statistics were used to characterize the cohort. Microbiome analysis was performed in R. Most analyses were performed using the open-source package MGSAT, which wraps several R packages to perform -omics analyses (https://github.com/andreyto/mgsat). Figures were generated with the R package ggplot2 (16). Associations were considered significant if the *p* or q value (as appropriate) was <0.05. Additional details describing the tests and parameters are delineated in the supplementary material (see Supplementary Digital Content 1, http://links.lww.com/CTG/A37).

Significant associations between clinical, endoscopic, or histologic metadata and bacterial taxa at the operational taxonomic unit and genus levels were assessed using the R package DESeq2 (17). Reported q values are the result of a Wald test with the Benjamini-Hochberg correction (18) applied to adjust for multiple comparisons. Richness and alpha and beta diversity metrics were calculated with the R package vegan (19) at the OTU level. Beta diversity was assessed with the Bray-Curtis dissimilarity index, and the PermANOVA test as implemented in Adonis (20) was used to test for significant differences between overall microbial composition and metadata groupings. Richness was assessed by calculating the abundance-based S. chao (21) index and by estimating the number of OTUs in each sample (hereafter referred to as S.obs). Alpha diversity was assessed using Hill numbers N1 and N2, which are, respectively, the exponential of the Shannon index and the inverted Simpson index (22). Generalized linear models were fit to test for significant associations between metadata categories and richness/alpha diversity indexes.

### Ethics

This study was approved by the Vanderbilt University Institutional Review Board (protocol number 151341).

## RESULTS

### Demographics and clinical parameters

In all, 49 children were enrolled in this study, and 4 were excluded from analysis for concomitant eosinophilic gastritis or eosinophilic gastroenteritis. Of the 45 children included, 15 (33%) had active EoE, 11 (24%) had inactive EoE, and 19 (42%) were non-EoE controls. The study groups were comparable for age and ethnicity, with the median (interquartile range) age of the cohort being 11 (10–15) years, and comprised predominantly of whites (84%). Most children with EoE were male compared with the non-EoE controls (85% vs 37%; *P* = 0.001), and the proportion of children with inactive EoE was comparable to that of proportion of children with active EoE (90% vs 80%; *P* = 0.49). Abdominal pain (62%) and dysphagia (27%) were 2 of the most common indications for EGD. A significantly higher proportion of children with active EoE presented with dysphagia compared with non-EoE controls (47% vs 11%; *P* = 0.02). Allergic rhinitis (53%) and environmental allergies (42%) were the most common atopic comorbidities. At the time of EGD, 33 (73%) were on proton pump inhibitors, 15 (33%) on oral antihistaminics, 8 (18%) on nasal topical steroids for allergic rhinitis, 9 (20%) on inhaled steroids for asthma, 6 (13%) on inhaled and swallowed steroids for EoE, and 2 (4%) were on swallowed topical steroids (a slurry) for EoE. None of the participants were on dietary elimination therapy for EoE. The medication exposure was comparable among children with EoE and non-EoE controls. A significantly higher proportion of children with inactive EoE were on inhaled/swallowed steroids when compared with children with active EoE (45% vs 7%; P50.02). The children with active EoE had the highest EREFS 2 (1–7) and highest EoEHSS 0.45 (0.09–1) (both presented as median [range]). The results are summarized in Table 1.

**Table 1.**
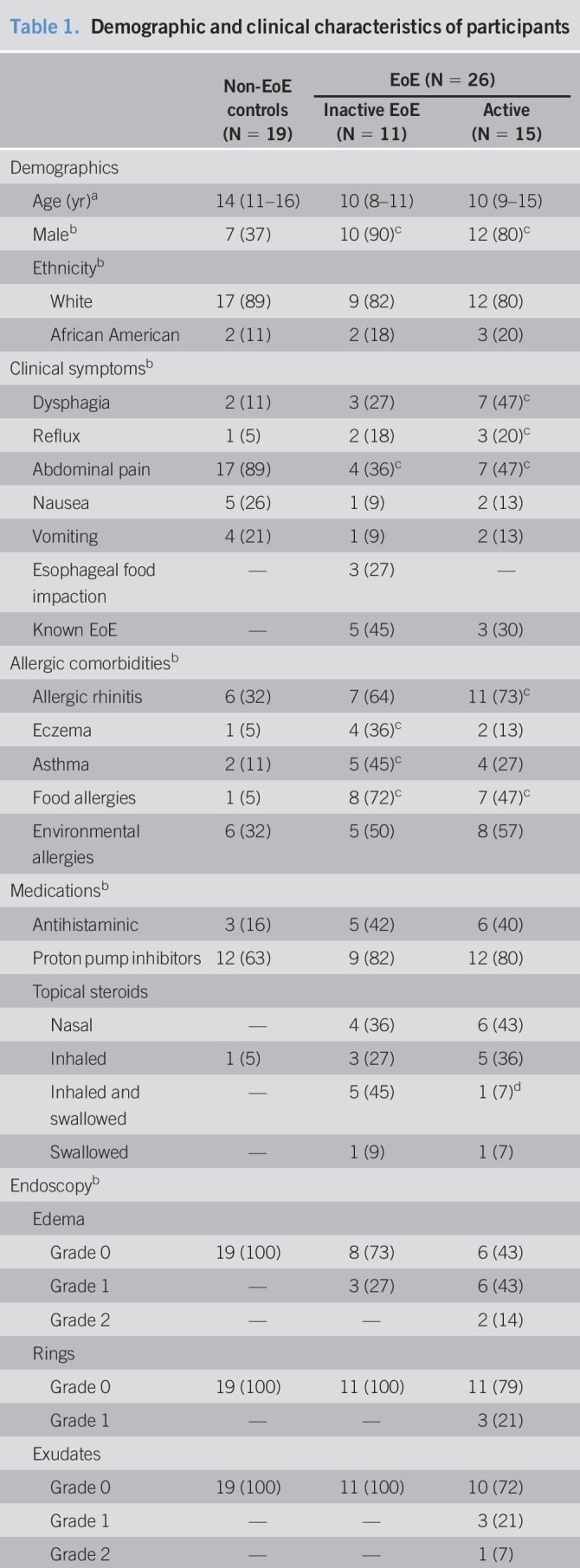

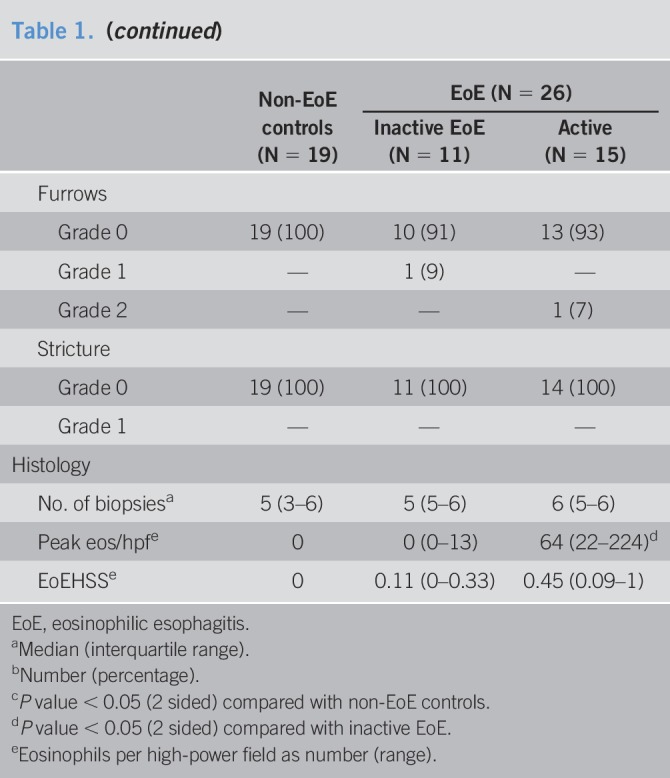
Demographic and clinical characteristics of participants

### Salivary microbiome composition in children with EoE and non-EoE controls

**Figure 1.**
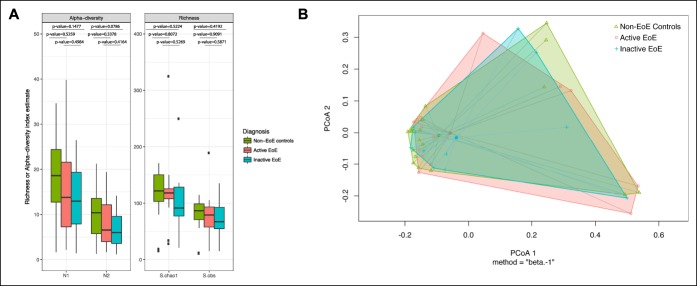
Richness and alpha and beta diversity of the salivary microbiome in children with active EoE, children with inactive EoE, and non-EoE control children. (**a**) The salivary microbiome alpha diversity (measured as Hill numbers N1, the exponential of the Shannon index, and N2, the inverted Simpson index) and richness (measured as S.chao1 and S.obs, the estimated number of OTUs) were compared between children with active EoE, inactive EoE, and those without EoE. Although richness and alpha diversity were highest in children without EoE and lowest in children with inactive EoE, these differences were not significant. (**b**) PcoA plot based on beta diversity estimated with Bray-Curtis dissimilarities in saliva microbiome communities. At a broad level, microbial community composition was similar between patients with active EoE, patients with inactive EoE, and healthy controls. EoE, eosinophilic esophagitis; PcoA, principal coordinates analysis.

Our 16S rRNA sequencing resulted in a median (interquartile range) of 15,960 (9,725–19,120) bacterial sequence reads per sample retained after data processing and quality control. On average per sample, the most abundant phyla were Firmicutes (35%), Bacteroidetes (31%), Proteobacteria (23%), Actinobacteria (5%), and Fusobacteria (4%). At the genus level, the most abundant genera were *Prevotella* (25%), *Streptococcus* (13%), *Veillonella* (11%), *Moraxella* (8%), *Haemophilu*s (5%), and *Neisseria* (4%). There was a nonstatistically significant trend toward higher microbial richness and alpha diversity in non-EoE controls compared with children with active EoE (*P* > 0.07) (Figure 1a). Beta diversity testing revealed that broad microbial composition was comparable (Bray-Curtis index; *P* = 0.93) among the 3 groups (Figure 1b).

### Composition of salivary microbiome is altered in children with EoE compared with non-EoE controls

**Figure 2.**
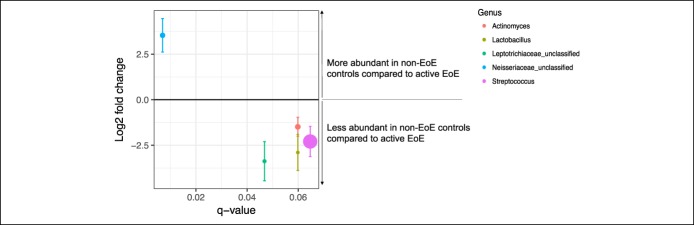
Differences of the salivary microbiome of children with active EoE compared with non-EoE controls. All displayed values were calculated within the DESeq2 package, where we compared genera abundance in active EoE children with non-EoE controls, with age, sex, ethnicity, and medication exposure added to the model as covariates. On the x-axis is displayed the q value for the tested genus; only putatively significant genera with q values < 0.1 are shown. On the y-axis is displayed the log_2_ fold abundance change for that genus when active EoE and non-EoE samples were compared. Error bars show the standard error of the log_2_ fold change. Log_2_ fold changes >0 indicate that a genus was more abundant in non-EoE controls compared with active EoE samples; log_2_ fold changes <0 indicate that a genus was less abundant in non-EoE controls compared with active EoE samples. The size of each point represents the base mean count of that genus; a larger point size indicates greater abundance. Neisseriaceae_unclassified was more abundant in non-EoE control children, whereas *Actinomyces, Lactobacillus, Leptotrichiaceae*_unclassified, and *Streptococcus* were less abundant in non-EoE control children, when samples were compared with children with active EoE. EoE, eosinophilic esophagitis.

**Figure 3.**
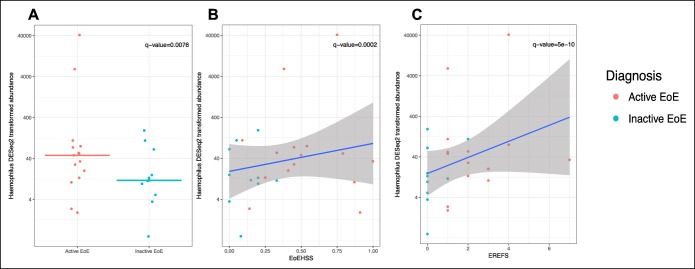
Relationship between the salivary microbiome and EoE activity indexes in children with EoE. All dots displayed are the *Haemophilus* abundance in each sample, transformed by the DESeq2 package. There was an overall trend of higher *Haemophilus* abundance being associated with active EoE and more severe disease. (**a**) Differential abundance of *Haemophilus* in the saliva microbiome of inactive EoE and inactive EoE samples. Lines represent the median transformed *Haemophilus* abundance per each diagnosis. (**b** and **c**) Differential abundance of *Haemophilus* in the saliva microbiome of inactive EoE and inactive EoE samples and their association with the EoEHSS and EREFS. EoE, eosinophilic esophagitis; EREFS, Eosinophilic Esophagitis Endoscopic Reference Score.

We conducted refined analyses to investigate whether specific taxa were differentially abundant. Pairwise analyses, adjusted for potential confounders (age, sex, ethnicity, and medication exposure), to examine the directionality and magnitude of observed differentially abundant taxa revealed that children with active EoE had significantly lower relative abundance of Leptotrichiaceae_unclassified (base mean = 12.9726, log_2_ fold change = −3.3750, q value= 0.04) and trended to have lower abundances of *Actinomyces* (base mean = 99.8522, log_2_ fold change = −1.4859, q value = 0.05), *Lactobacillus* (base mean = 8.2011, log_2_ fold change = −2.8941, q value = 0.05), and *Streptococcus* (base mean = 2,543.5310, log_2_ fold change = −2.2904, q value = 0.06) compared with non-EoE controls (Figure 2). The non-EoE controls had significantly higher relative abundance of *Neisseriaceae*_unclassified compared with children with active EoE (base mean = 75.0051, log_2_ fold change = 3.5347, q value = 0.006) (Figure 2). A significantly higher relative abundance of *Haemophilus* (base mean = 1858.625, log_2_ fold change = −3.111, q value = 0.008) was observed when children with active EoE were compared with children with inactive EoE (Figure 3a).

### Salivary microbiome in children with EoE correlates with validated EoE activity indexes

In children with EoE, the relative abundance of *Haemophilus* (base mean = 1858.625, log_2_ fold change = 0.02, q value = 0.0002) at the genus level and a *Pasteurellaceae*_unclassified OTU (base mean = 2,977.989, log_2_ fold change = 2.058e-02, q value < 0.001), a *Haemophilus* OTU (base mean = 291.029, log_2_ fold change = 1.897e-02, q value = 0.001), and a *Lactobacillus* OTU (base mean = 3.127, log_2_ fold change = 1.239e-02, q value = 0.03) significantly increased with increasing density of eosinophilic infiltration (with log_2_ fold change given per unit of change of the infiltration density, eos/hpf). Similarly, the relative abundance of *Haemophilus* had a significantly positive correlation with esophageal mucosal abnormalities as assessed by the EREFS (base mean = 1942, log_2_ fold change = 1.4332, q value = 5.370e-10) and increasing histopathologic severity as assessed by the EoEHSS (base mean = 2014.595, log_2_ fold change = 5.8667, q value < 0.001) (Figure 3b,c). Neither microbial richness nor alpha diversity significantly correlated with the increasing density of eosinophilic infiltration, EREFS, or EoEHSS (see Figure 1, Supplementary Digital Content 2, http://links.lww.com/CTG/A38).

### Clusters of salivary microbiome correlate with the histologic changes in EoE

**Figure 4.**
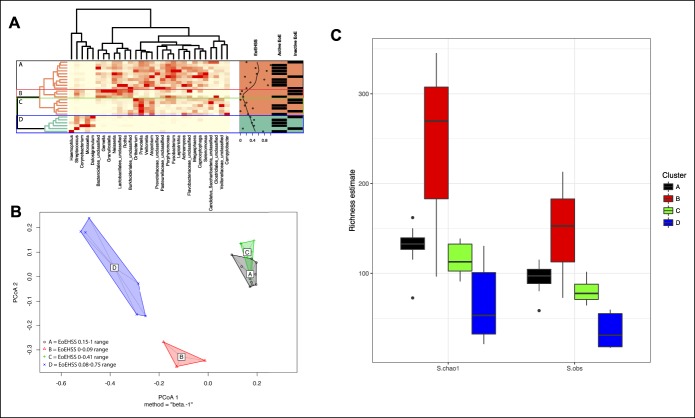
Relationship between salivary microbiome clusters and EoEHSS scores in children with EoE. (**a**) Heatmap showing the top 30 most abundant genera, with samples (rows) clustered based on the Bray-Curtis dissimilarity metric between the relative genus abundance profiles, and the clinical covariates such as the EoEHSS severity score and the active/inactive EoE status plotted for each sample. Genera names are shown on the bottom; stronger red color on the heatmap indicates higher genus abundance. Four microbial community clusters, termed A-D, are labeled on the left. On the right is displayed the EoEHSS score for that patient, along with whether the sample was from a child with active or inactive EoE. The EoEHSS is associated with the broad microbial clusters A-D according to the Kruskal-Wallis test as described in the text. (**b**) PcoA plot showing beta diversity (calculated with Bray-Curtis dissimilarities) at the OTU level. The samples were labeled according to their membership in the clusters A-D as derived from the heatmap. Samples from clusters D and B were highly dissimilar to all the others, whereas clusters A and C were similar to each other. (**c**) The microbial richness for the clusters based on the heatmap, as calculated with S.Chao1 and S.obs (estimated number of OTUs). Microbial richness was highest in samples from cluster A and lowest in those from cluster D. EoE, eosinophilic esophagitis; PcoA, principal coordinates analysis.

Using a heatmap based on the top 30 most abundant genera, we delved deeper into the ongoing potential mechanistic processes that could unravel the relationship between alterations in the salivary microbiome and disease status in children with EoE. The EoEHSS correlated with 4 broad salivary microbial communities representing clades in the unsupervised hierarchical clustering of the taxonomic abundance profiles, identified as clusters A–D (Figure 4a); a Kruskal-Wallis test revealed significant differences in the EoEHSS by cluster designation (*P* = 0.0108) (see Figure 2, Supplementary Digital Content 2, http://links.lww.com/CTG/A39). Cluster A corresponded with the highest EoEHSS scores (range: 0.15–1) and was defined by higher abundances of a broad range of taxa, including *Gemella*, *Neisseria*, *Rothia*, *Prevotella*, and *Veillonella*. Cluster B was associated with the lowest EoEHSS scores (range: 0–0.09) and was also characterized by high abundances of *Streptococcus*, *Gemella*, *Granulicatella*, *Neisseria*, and *Rothia*, but unlike those from cluster A, *Prevotella* and *Veillonella* abundance was low. Cluster C was linked to intermediate EoEHSS scores (range: 0–0.41) and higher abundances of *Oribacterium*, *Prevotella*, *Veillonella*, and *Atopobium*. Cluster D was associated with higher EoEHSS scores (range: 0.08–0.75) and was defined by higher abundance of *Haemophilus*, *Streptococcus*, *Corynebacterium*, *Moraxella*, and *Dolosigranulum*. To further validate the presence of these microbial clusters observed at the genus level, we conducted additional analyses at the OTU level. At the OTU level, when samples were split by these clusters, the overall microbial communities were significantly distinct between the 4 clusters (PermANOVA *P* = 0.0002); however, samples from clusters A and C were relatively similar (Figure 4b). As would be predicted from the heatmap, samples from cluster D had the lowest microbial richness (Figure 4c). The salivary microbiome clusters did not correlate with any of the demographic and clinical metadata or the EREFS.

### Relationship between medication exposure and the composition of salivary microbiome

As PPIs, inhaled/swallowed, and/or swallowed topical corticosteroids are routinely used in management of EoE, we examined the effect of these medications on the salivary microbiome composition. In total, 33 of the children were on PPIs and 12 were not on PPIs. The PPI use was nonsignificantly lower in non-EoE controls (63%) compared with those with inactive EoE (82%) or active EoE (80%). The PPI use was associated with a higher abundance of *Streptococcus* (base mean = 2,687.9599, log_2_ fold change = 3.1740, q value = 4.28e-05), *Corynebacterium* (base mean = 77.8230, log_2_ fold change = 3.0577, q value = 0.001), and *Rothia* (base mean = 38.4750, log_2_ fold change = 1.2574, q value = 0.01). Although the PPI use was not significantly associated with a difference in microbial richness or alpha or beta diversity, the richness and alpha diversity tended to be lower in children who were using PPIs (all *P* > 0.20). If analysis was restricted to children with EoE (active and inactive), PPI use (N = 21) compared with no PPI use (N = 5) was not significantly associated with any microbiome changes. Similarly, the use of inhaled and swallowed corticosteroids (N = 6) and/or swallowed corticosteroids (N = 2) in children with EoE (active and inactive) was not significantly associated with differential abundance of any taxa or with changes in the microbial richness or alpha or beta diversity (all relevant *P*- or q values > 0.30).

## DISCUSSION

In this case-control study, we used 16S rRNA gene sequencing to characterize the composition of the salivary microbiota of children with EoE compared with non-EoE controls. We observed nonsignificant but notable differences in the overall salivary microbiome diversity and composition between children with EoE and non-EoE controls. In addition, in children with EoE, the richness of some distinguishing species positively correlated with validated disease activity indexes. Through an exploratory analysis involving 30 of the most common genera, we were able to discern salivary microbiome profiles which correlated with the intensity and severity of histologic changes observed in the esophageal biopsies—the current gold standard for the diagnosis and monitoring of EoE. These novel findings enhance our understanding of the role of the microbiome in EoE. In particular, it highlights the role of salivary microbiome in pathobiology of EoE.

The relative abundance of salivary *Haemophilus* was positively correlated with the validated EoE activity indexes after adjusting for potential confounders. Enrichment of *Haemophilus* in the hypopharyngeal region has been associated with an increased risk of other conditions characterized by eosinophil-mediated allergic inflammation such as recurrent wheezing and asthma in children (23). Similarly, an abundance of *Haemophilus* in the sinonasal cavity has been demonstrated in patients with chronic rhinosinusitis (24,25), and an abundance of *Haemophilus* in the sputum has been associated with bacterial exacerbations of chronic obstructive pulmonary disease (26). At the genus level, children with active EoE had a lower relative abundance of *Leptotrichiaceae*_unclassified, *Actinomyces*, *Lactobacillus*, and *Streptococcus* compared with non-EoE controls. *Actinomyces* and *Streptococcus* are among the most abundant genera in saliva collected from adults (27,28). It is unclear whether the decreased abundance of these genera in our cohort of children with EoE is related to their age and/or their disease. Finally, there was a nonsignificant trend toward highest microbial richness and alpha diversity in non-EoE controls compared with children with EoE, suggesting that decreased salivary microbial richness/alpha diversity could be indicative of EoE. Decreased richness of the gut microbiome has previously been associated with other disease states (29), and further research in larger cohort is warranted to investigate whether a decrease in microbial richness could be a predictor or mediator of EoE.

There was a modest overlap between the salivary microbiome composition in our cohort of children with EoE and previously published reports describing the esophageal microbiome in patients with EoE. Benitez et al. (10) evaluated the differences between the oral (collected by swab) and esophageal (in the esophageal biopsies) microbiomes in children with and without EoE and observed a shift in the relative abundance of Proteobacteria including *Neisseria* and *Corynebacterium* in children with active EoE, whereas Firmicutes (including *Streptococcus* and *Atopobium*) were enriched among non-EoE controls. They reported a modest correlation between oral and esophageal microbiomes for Bacteroides, Firmicutes, and Proteobacteria species. In another study, Harris et al. (11) observed significantly increased abundance of Proteobacteria (mostly *Haemophilus*) in esophageal mucosal samples collected from children and adults with EoE and a decrease in the extent of Firmicutes in patients with active EoE compared with inactive EoE and non-EoE controls. This is consistent with our current understanding that the esophageal microbiome can be broadly similar to the oral microbiome because both contain an abundance of anaerobes and a high ratio of Firmicutes and Bacteroidetes, and that the oral microbiome can shape the esophageal microbiome through migration of oral bacteria via swallowed or salivary secretions (30,31). Taken together, our findings suggest that although the oronasopharyngeal area and esophagus are anatomically 2 distinct locations, an allergen-mediated eosinophilic inflammation in the esophagus may be linked to oral and salivary dysbiosis. Our findings also raise important mechanistic questions regarding the role of salivary *Haemophilus* in development of EoE.

This study has limitations. One of the major limitations is a relatively small sample size, which probably resulted in nonsignificant statistical trends. However, these results lay foundation for a larger and sufficiently powered study in the future. Although none of our participants were on dietary elimination therapy alone for EoE, some of them were avoiding foods not specifically as a part of their EoE therapy but for reasons such as texture issues, disliking certain foods, partially avoiding foods such as avoiding milk but consuming yogurt and cheese, empirically eliminating gluten for their gastrointestinal symptoms, and skin prick test–based dietary avoidances in children with known EoE. As a result, we were unable to account for the effect of variability in diet on the composition of salivary microbiome. However, because the participants were nil per os for at least 6 hours before providing saliva samples (per protocol for their EGD), we were able to minimize any immediate effect of diet on the salivary microbiome. Next, the saliva samples were collected as spit, a commonly used collection method, and preprocessed before microbial analyses. Although there is lack of consensus on the optimal method(s) to collect and preprocess saliva for microbial analysis, it is unclear how these steps may have affected our saliva sequencing analysis. However, it is reassuring that the salivary microbiome profiles are minimally affected by commonly used collection methods or DNA extraction protocol (32). This approach needs to be validated in larger and distinct populations before adoption into clinical practice. Our cohort predominantly consisted of males and whites. Although this is consistent with our current understanding of the age and sex distribution of patients with EoE, the results may have to be cautiously applied to patients with EoE who are in other age groups, female, and/or belong to other ethnicities. Finally, this study remains descriptive; future *in vivo* experiments would be necessary to establish a causal relationship between alterations in the salivary microbiome and EoE.

Despite these limitations, this study is among the first few studies to characterize the salivary microbiome in children and the first to our knowledge to characterize the salivary microbiome in children with EoE and compare it with non-EoE controls. Our participants were consecutively enrolled, allowing us to eliminate a selection bias. The saliva samples were collected within a narrow period, minimizing any influence of circadian rhythm on the composition of salivary microbiome. We collected and analyzed comprehensive clinical metadata (e.g., history of atopic comorbidities, including atopic non-EoE controls, and local corticosteroid use of all types), allowing us to assess how clinical factors influenced the relationship between the salivary microbiome and EoE. All the EGDs were performed by a single investigator, and all the biopsies were examined by a single blinded pathologist; this ensured a high level of uniformity of evaluating the EREFS and EoEHSS, respectively. Finally, we were able to identify salivary microbiome profiles associated with categories of the EoEHSS, indicating that variations in the salivary microbial communities may in general be stratified, and functional analysis might allow us to understand the contribution of microbial communities to EoE pathobiology.

In conclusion, saliva is a biofluid potentially rich in diagnostic indicators for both oral and systemic disorders. The composition of the salivary microbiome in children with EoE seems to be altered compared with that of non-EoE controls, and a relative abundance of *Haemophilus* may have a role in the pathobiology of EoE. Furthermore, alterations in the salivary microbiome could serve as a practical and noninvasive approach to identify and monitor EoE status (active or inactive). The exact mechanisms underlying the complex interactions between the salivary microbiome, innate immune system, an allergen specific inflammatory response, and esophageal inflammation warrants further research.

## DATA ACCESS

Sequencing data has been published at the National Center for Biotechnology Information (NCBI) Sequence Read Archive (SRA) under BioProject PRJNA532939; runs SRR8903593 - SRR8903637.

## CONFLICTS OF INTEREST

**Guarantor of the article:** Girish Hiremath, MD, MPH.

**Specific author contributions:** Study conception and design: G.H., H.C., S.A., and S.R.D. Collecting data and analyzing biopsies and saliva samples: G.H., M.H.S., H.C., S.V.R., and S.R.D. Generation, analysis, and interpretation of salivary microbiome data: G.H., M.H.S., H.H.B., A.T., S.V.R., and S.R.D. Critical revisions of the manuscript: G.H., M.H.S., H.H.B., H.C., S.A., A.T., S.V.R., and S.R.D.

**Financial support:** This study was funded by the American College of Gastroenterology Clinical Research Award. G.H. is supported by the American College of Gastroenterology Junior Faculty Career Development Award, Vanderbilt University Turner Hazinski award, Vanderbilt University Katherine Dodd Faculty Scholar program, and the Consortium of Eosinophilic Gastrointestinal Disease Researchers (U54 AI117804) training award. Consortium of Eosinophilic Gastrointestinal Disease Researchers (CEGIR) is part of the Rare Disease Clinical Research Network, an initiative of the Office of Rare Diseases Research, National Center for Advancing Translational Sciences, and is funded through collaboration between the National Institute of Allergy and Infectious Diseases, the National Institute of Diabetes and Digestive and Kidney Diseases, and the National Center for Advancing Translational Sciences. The CEGIR is also supported by patient advocacy groups including the American Partnership for Eosinophilic Disorders, Campaign Urging Research for Eosinophilic Diseases, and Eosinophilic Family Coalition. S.D. is supported by the Vanderbilt Institute for Clinical and Translational Research grant support (NIH/NCATS UL1 TR000445, U54 RR24975). S.R.D. is also supported by NIH-funded Tennessee Center for AIDS Research (P30 AI110527) and U19AI095227. Content is solely the responsibility of the authors and does not represent official views of the CDC and the NIH.

**Potential competing interests:** A.T. is employed by MedImmune, the biologics arm of AstraZeneca, and owns AstraZeneca stock.

**Study Highlights**

**WHAT IS KNOWN**

✓ EoE is a chronic, food and/or aeroallergen-mediated, eosinophil-predominant inflammatory condition affecting the esophagus.
✓ Little is known about the role of the microbiome in EoE.
✓ To date, the composition of the salivary microbiome in children with EoE and its relationship with disease activity status have not been studied.

**WHAT IS NEW HERE**

✓ The composition of the salivary microbiome is altered in children with EoE compared with non-EoE controls.
✓ In children with EoE, abundance of salivary *Haemophilus* positively correlates with validated endoscopic and histologic disease activity indexes.

**TRANSLATIONAL IMPACT**

✓ This study provides novel insights into the role of the salivary microbiome in EoE pathobiology.
✓ Alterations in the salivary microbiome may hold potential to serve as a noninvasive marker to monitor EoE activity in children.

## Supplementary Material

SUPPLEMENTARY MATERIAL
