# Supplementary material for: The Salivary Microbiome Is Altered in Children With Eosinophilic Esophagitis and Correlates With Disease Activity
Source: Clin Transl Gastroenterol. 2019 May 20;10(6):e00039. doi: 10.14309/ctg.0000000000000039 (PMC6613866; doi:10.14309/ctg.0000000000000039)
Supplement: SUPPLEMENTARY MATERIAL [file ct9-10-e00039-s001.docx]

**Title: The Salivary Microbiome is Altered in Children with Eosinophilic Esophagitis and it Correlates with Disease Activity.**

**Short title:** Salivary Microbiome in Eosinophilic Esophagitis

**Authors:**

Girish Hiremath, MD MPH

Division of Pediatric Gastroenterology, Hepatology and Nutrition

2200 Children’s way, 10^th^ Floor

Vanderbilt University Medical Center, Nashville TN 37232

Meghan H. Shilts, MHS MS

Division of Infectious Diseases

Vanderbilt University Medical Center

1211, 21st Avenue South

S2108 Medical Center North, Nashville, TN 37232

Helen H. Boone, BAS

Division of Infectious Diseases

Vanderbilt University Medical Center

1211, 21st Avenue South

S2108 Medical Center North, Nashville, TN 37232

Hernan Correa, MD

Division of Pediatric Pathology

2200 Children’s way, 11^th^ Floor

Vanderbilt University Medical Center, Nashville, TN 37232

Sari Acra, MD MPH

Division of Pediatric Gastroenterology, Hepatology and Nutrition

2200 Children’s way, 10^th^ Floor

Vanderbilt University Medical Center, Nashville TN 37232

Andrey Tovchigrechko, PhD

Research Bioinformatics

MedImmune

1 MedImmune Way, Gaithersburg, MD 20878

Seesandra V. Rajagopala, PhD

Division of Infectious Diseases

Vanderbilt University Medical Center

1211, 21st Avenue South

S2108 Medical Center North, Nashville, TN 37232

Suman R. Das, PhD

Division of Infectious Diseases

Vanderbilt University Medical Center

1211, 21st Avenue South

S2108 Medical Center North, Nashville, TN 37232

**Corresponding authors:**

**Suman R. Das**

Email: [suman.r.das@vanderbilt.edu](mailto:suman.r.das@vanderbilt.edu)

Phone: 615-322-0322

**Seesandra V. Rajagopala**

E-Mail: [s.v.rajagopala@vanderbilt.edu](mailto:s.v.rajagopala@vanderbilt.edu)

Phone: 615-322-2419

**Address:**

Division of Infectious Diseases

Vanderbilt University Medical Center

1211, 21st Avenue South

S2108 Medical Center North

Nashville, TN 37232

**Word count:**

Manuscript: 3361

**Tables:** 1

**Figures:** 4

**Supplementary material:** 1

**SUPPLEMENTARY MATERIALS AND METHODS**

***Data processing and statistical analysis***

Reads were processed by following the mothur MiSeq SOP (www.mothur.org/wiki/MiSeq_SOP) as of August 4, 2017.^1^ Reads were aligned against the SILVA database release 128^2^ and taxonomy was assigned using the RDP database version 11.^3^ Operational taxonomic units (OTUs) were clustered at 97% similarity.

Descriptive statistics were used to characterize the cohort. Microbiome analysis was performed in R. The majority of the analyses were done using the open source package MGSAT, which wraps several R packages in order to perform -omics analyses [https://github.com/andreyto/mgsat]. Figures were generated with the R package ggplot2.^4^ Associations were considered significant if the p- or q-value (as appropriate) was < 0.05.

Significant associations between clinical, endoscopic, or histologic metadata and bacterial taxa at the OTU and genus levels were assessed using the R package *DESeq2*.^5^ To reduce the penalty associated with multiple testing and to remove likely non-informative features, prior to DESeq2 analysis, taxa with an average relative abundance < 0.0005 were aggregated into a group called “other” and this group was included during testing but was otherwise ignored. For the DESeq2 analyses, we built both unadjusted and adjusted models. Our adjusted models included covariates expected *a priori* to influence the salivary microbiome: age, gender, ethnicity, and exposure to medications. Reported q-values are the result of a Wald test with the Benjamini-Hochberg correction^6^ applied to adjust for multiple comparisons.

Richness and alpha- and beta-diversity metrics were calculated with the R package *vegan^7^* at the OTU level; all OTUs regardless of abundance were included. To control for differences in sequencing depth per sample, samples were randomly rarefied to the minimum sample read count and then each richness, alpha- or beta-diversity index was calculated. For each index, this process was repeated 400 times and results were averaged. Beta diversity was assessed with the Bray-Curtis dissimilarity index and the PermANOVA test as implemented in *Adonis*^8^ was used to test for significant differences between overall microbial composition and metadata groupings. Richness was assessed by calculating the abundance-based S. chao^9^ index and by estimating the number of OTUs in each sample (hereafter referred to as S.obs). Alpha diversity was assessed using Hill numbers N1 and N2, which are, respectively, the exponential of the Shannon index and the inverted Simpson index.^10^ Generalized linear models were fit to test for significant associations between metadata categories and richness/alpha-diversity indices.

**REFERENCES**

1 Kozich, J. J., Westcott, S. L., Baxter, N. T., Highlander, S. K. & Schloss, P. D. Development of a dual-index sequencing strategy and curation pipeline for analyzing amplicon sequence data on the MiSeq Illumina sequencing platform. *Applied and environmental microbiology* **79**, 5112-5120, doi:10.1128/aem.01043-13 (2013).

2 Pruesse, E. *et al.* SILVA: a comprehensive online resource for quality checked and aligned ribosomal RNA sequence data compatible with ARB. *Nucleic acids research* **35**, 7188-7196, doi:10.1093/nar/gkm864 (2007).

3 Cole, J. R. *et al.* The Ribosomal Database Project: improved alignments and new tools for rRNA analysis. *Nucleic acids research* **37**, D141-145, doi:10.1093/nar/gkn879 (2009).

4 Wickham, H. *ggplot2: elegant graphics for data analysis*. (Springer, 2009).

5 Love, M. I., Huber, W. & Anders, S. Moderated estimation of fold change and dispersion for RNA-seq data with DESeq2. *Genome Biol* **15**, 550, doi:10.1186/s13059-014-0550-8 (2014).

6 Benjamini, Y. & Hochberg, Y. Controlling the false discovery rate: a practical and powerful approach to multiple testing. *J R Stat Soc Series B Stat Methodol* **57**, 289-300, doi:10.2307/2346101 (1995).

7 Oksanen, J. *et al.* vegan: Community Ecology Package. R package version 2.0-10. (2014).

8 Anderson, M. J. A new method for non-parametric multivariate analysis of variance. *Austral Ecol* **26**, 32-46, doi:10.1111/j.1442-9993.2001.01070.pp.x (2001).

9 Chao, A. Estimating the population size for capture-recapture data with unequal catchability. *Biometrics* **43**, 783-791 (1987).

10 Hill, M. O. Diversity and evenness: a unifying notation and its consequences. *Ecology* **54**, 427-432, doi:10.2307/1934352 (1973).

**GUARANTOR OF THE ARTICLE**

Girish Hiremath, MD MPH

**AUTHOR CONTRIBUTIONS**:

Study conception and design: GH, SA, HC, SRD

Collecting data, analyzing biopsies and saliva samples: GH, MHS, HC, SVR, SRD

Generation, analysis, and interpretation of salivary microbiome data: GH, MHS, HB, AT, SVR, SRD

Critical revisions of the manuscript: GH, MS, HB, AT, HC, SA, SVR, SRD.

**FINANCIAL SUPPORT**

This study was funded by the American College of Gastroenterology Clinical Research Award. G.H. is supported by American College of Gastroenterology Junior Faculty Career Development Award, Vanderbilt University Turner Hazinski award, Vanderbilt University Katherine Dodd Faculty Scholar program, and the Consortium of Eosinophilic Gastrointestinal Disease Researchers (U54 AI117804) training award. Consortium of Eosinophilic Gastrointestinal Disease Researchers (CEGIR) is part of the Rare Disease Clinical Research Network, an initiative of the Ofﬁce of Rare Diseases Research, National Center for Advancing Translational Sciences, and is funded through collaboration between the National Institute of Allergy and Infectious Diseases, the National Institute of Diabetes and Digestive and Kidney Diseases, and the National Center for Advancing Translational Sciences. The CEGIR is also supported by patient advocacy groups including the American Partnership for Eosinophilic Disorders, Campaign Urging Research for Eosinophilic Diseases, and Eosinophilic Family Coalition. Vanderbilt Institute for Clinical and Translational Research grant support (NIH/NCATS UL1 TR000445, U54 RR24975). SD is also supported by NIH-funded Tennessee Center for AIDS Research (P30 AI110527) and U19AI095227. Content is solely the responsibility of the authors and does not represent official views of the CDC and the NIH.

**POTENTIAL COMPETING INTEREST**

Andrey Tovchigrechko is employed by MedImmune, the biologics arm of AstraZeneca, and owns AstraZeneca stock.
