# Supplementary figures and images for: The Salivary Microbiome Is Altered in Children With Eosinophilic Esophagitis and Correlates With Disease Activity
Source: Clin Transl Gastroenterol. 2019 May 20;10(6):e00039. doi: 10.14309/ctg.0000000000000039 (PMC6613866; doi:10.14309/ctg.0000000000000039)

Supplementary Figure 1:

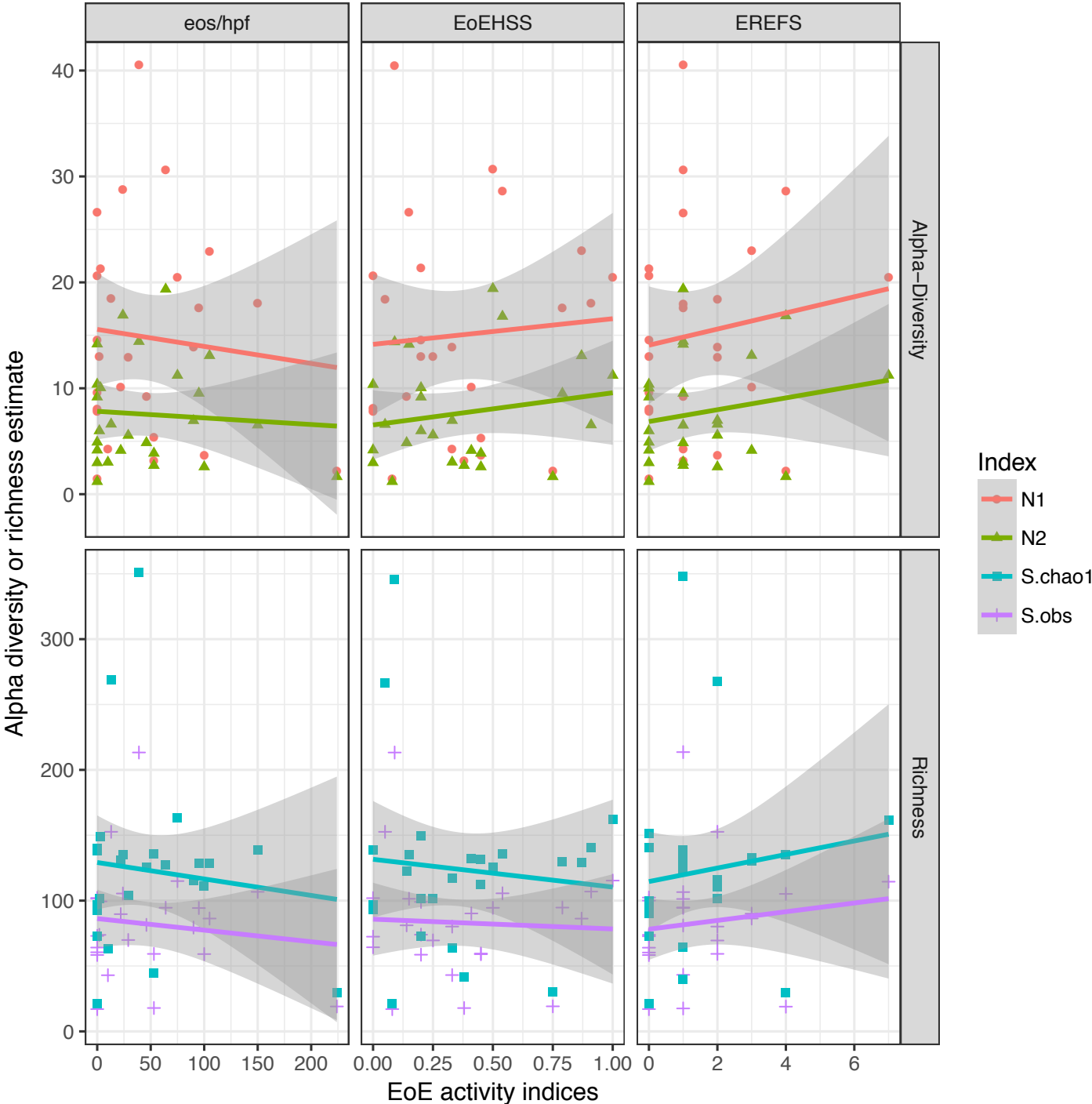

Supplement: SUPPLEMENTARY MATERIAL [file ct9-10-e00039-s002.pdf]

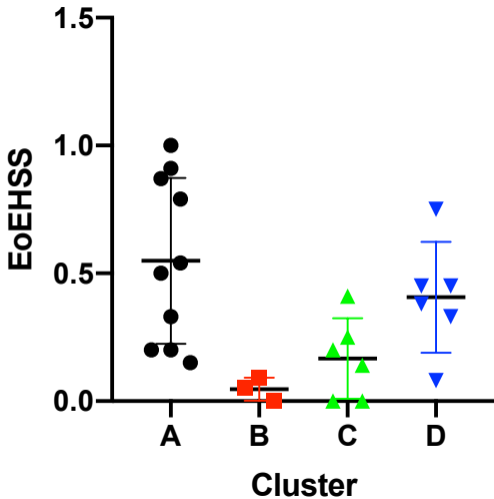

Supplement: SUPPLEMENTARY MATERIAL [file ct9-10-e00039-s003.pdf]
